# Study of the interaction between cardiometabolic index and inflammatory index on the risk of prostate cancer development

**DOI:** 10.3389/fimmu.2025.1591879

**Published:** 2025-07-17

**Authors:** Yunyun Xiao, Bei Tang, Jueqi Wang, Zhiruo Cai, Hengqing An, Ning Tao

**Affiliations:** ^1^ School of Public Health, Xinjiang Medical University, Urumqi, China; ^2^ Department of Maternal and Child Health, School of Public Health, Peking University, Beijing, China; ^3^ Department of Urology, The First Affiliated Hospital of Xinjiang Medical University, Urumqi, China; ^4^ Xinjiang Clinical Medical Research Center for Urological and Male Reproductive System Diseases, Urumqi, China

**Keywords:** prostate cancer, cardiometabolic index, inflammation, neutrophillymphocyte ratio, platelet lymphocyte ratio, systemic inflammation response index, lymphocyte monocyte ratio

## Abstract

**Background:**

Prostate cancer (PCa) is an important cause of fatality in older men, with inflammation and metabolic disorders as risk factors for PCa. This study examined how systemic inflammation, measured by inflammatory indices, interacts with the cardiometabolic index (CMI), a marker of obesity and dyslipidemia, to influence the risk of developing PCa.

**Methods:**

This study consisted of 1,591 male patients recruited from the Department of Urology at the First Affiliated Hospital of Xinjiang Medical University between 2022 and 2024. Propensity score matching was employed to adjust the sample size, resulting in a final cohort of 149 PCa patients and 296 matched controls. Logistic regression models and restricted cubic spline (RCS) analyses were employed to evaluate the associations between CMI and various inflammatory indices (e.g., PIV, SIRI, PLR, NLR, LMR) with prostate cancer. Interaction tests were conducted to investigate the impact of the interplay between inflammatory indices and CMI on the risk of prostate cancer.

**Results:**

NLR, PLR, PIV, and SIRI were significantly positively associated with prostate cancer (PCa) risk, whereas LMR exhibited a significant negative association. The CMI was significantly associated with an elevated risk of prostate cancer (PCa) (OR = 1.97, 95% CI: 1.38~2.81). Restricted cubic spline (RCS) analysis revealed a nonlinear dose-response relationship between CMI and prostate cancer (PCa) risk, with the risk plateauing at CMI ≈ 0.65. Sensitivity analyses confirmed the robustness of these results. Significant interactions were observed between CMI and inflammatory indices, particularly NLR, PLR, and LMR, suggesting synergistic effects on prostate cancer (PCa) risk.

**Conclusions:**

The present study demonstrated that inflammation indicators and CMI exhibited a strong association with the risk of PCa. Furthermore, a significant interaction was observed between CMI and inflammation indicators. These findings provide a novel perspective for PCa risk prediction and prevention, suggesting that inflammation and metabolic status should be considered together when assessing PCa risk.

## 1 Introduction

Prostate cancer (PCa) is one of the most common cancers worldwide and the fifth leading cause of cancer deaths in men (1). According to Global Cancer Statistics 2022, PCa accounts for 7.3% of all cancer cases, with over 1.46 million new cases and more than 390,000 deaths reported (1). Indeed, PCa is commonly diagnosed in men aged 50 years and older (2). With the aging of the global population and shifts in lifestyle patterns (3), the incidence of PCa is projected to rise annually, thereby posing a significant threat to the health of elderly men (4).

The etiology of PCa is complex (2). Some studies have shown that inflammation is a key factor that influences the progression of PCa (5). To illustrate, inflammation impacts every step of tumorigenesis, such as tumor initiation, promotion, and metastatic progression (6). The inflammatory tumor microenvironment drives PCa development. As inflammation progresses, tumor cells recruit additional leukocytes, promoting angiogenesis, proliferation, vascular and tissue growth, and remodeling, ultimately leading to the development of PCa (5). In the tumor microenvironment, the number of immuno-inflammatory cells such as lymphocytes, monocytes, neutrophils, and platelets is significantly altered, and these changes may reflect the extent of cancer progression (7–10), which can help in the diagnosis and prognostic assessment of the tumor (11, 12). Combined inflammatory indices provide a holistic assessment of systemic inflammation compared to individual markers (e.g., neutrophil count, lymphocyte count, etc.), significantly enhancing disease prediction (13, 14). For instance, indices such as the pan-immune-inflammation value (PIV), neutrophil-lymphocyte ratio (NLR), platelet-lymphocyte ratio (PLR), systemic inflammation response index (SIRI), and lymphocyte-monocyte ratio (LMR) have demonstrated prognostic value across multiple cancers (15–17), and they have predictive value for the prognosis of PCa (18–23).

Numerous pro-inflammatory and anti-inflammatory molecules are recognized as secretory products of human adipocytes or are associated with adipose tissue (24). Wakabayashi et al. (2015) developed a novel marker known as the cardiometabolic index (CMI), which integrates key metabolic factors such as waist circumference, triglycerides, and high-density lipoprotein cholesterol (HDL-C). By combining lipid and obesity parameters into a simple and reproducible marker, CMI effectively reflects obesity and dyslipidemia (25). Subsequent research has validated CMI not only as a robust marker of obesity and metabolic syndrome but also as a superior diagnostic tool for visceral adiposity compared to conventional abdominal obesity indices (e.g., waist circumference, waist-to-hip ratio), demonstrating enhanced sensitivity in identifying early metabolic dysregulation (26, 27). While obesity is thought to be a chronic low-grade inflammatory state that is linked to PCa (28), CMI remains a relatively new area of research in the cancer field with many studies still in the exploratory stage. The association between CMI and prostate cancer risk has not yet been reported in the literature. Furthermore, existing studies have primarily focused on the correlation between inflammation and the cardiometabolic index (CMI), as well as the mediating role of inflammation in the relationship between CMI and chronic diseases among older adults (29–31). The exploration of the association between inflammation and CMI remains limited, and the impact of their interaction on prostate cancer risk has yet to be reported.

The present study investigates the effect of the interaction between the inflammatory index and CMI on the risk of PCa. This research aims to fill the gap in the field, enrich our understanding of the mechanisms underlying PCa development, and provide a foundation for more accurate risk prediction, screening, intervention, prevention, and treatment of PCa in the future.

## 2 Methods

### 2.1 Data source

**Figure 1:**
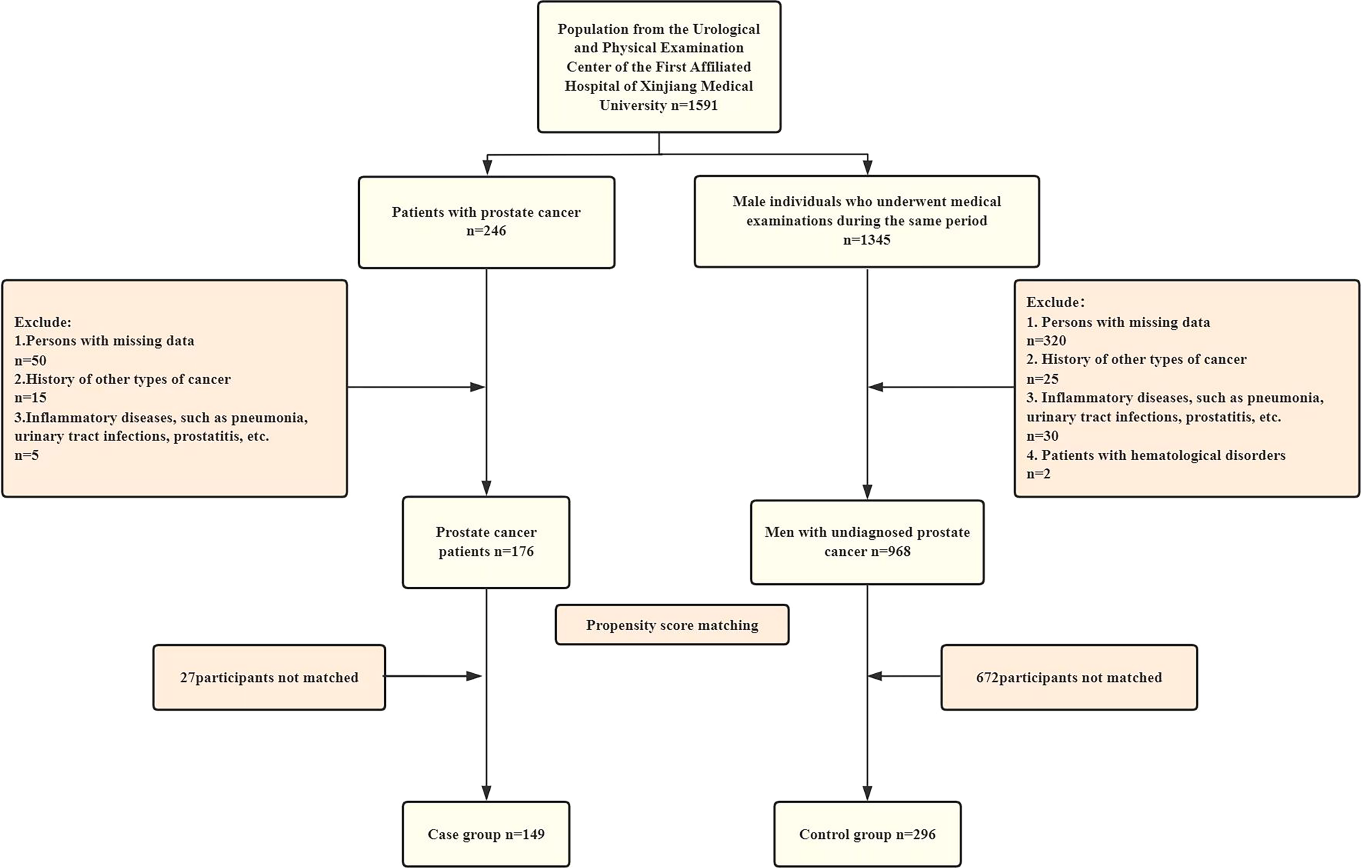
Flowchart of inclusion and exclusion of study participants.

This study collected clinical data from 1591 male patients who visited the Department of Urology at the First Affiliated Hospital of Xinjiang Medical University between 2022 and 2024. After applying the exclusion criteria, the sample included 246 patients with PCa diagnosed through prostate biopsy and puncture, along with 1,345 individuals who underwent examinations at a medical examination center. Propensity score matching (PSM) was employed to mitigate the impact of confounding variables. Nearest neighbor matching was performed with a 1:2 case-to-control ratio. Post-matching analysis revealed standardized mean differences (SMD) <0.1 for all covariates, demonstrating effective control of intergroup differences. The final matched cohort comprised 149 cases and 296 controls (**Figure 1**).

Inclusion criteria for case group: (1) patients first diagnosed with PCa by prostate biopsy from 2022 to 2024; (2) complete test data; (3) can read, understand and sign the informed consent form.

Exclusion criteria for case group: (1) PCa patients with a history of other types of cancer;(2) patients with inflammatory diseases, such as pneumonia, urinary tract infections, or prostatitis.

Inclusion criteria for control group:(1) male individuals who underwent physical examinations at the Physical Examination Center of the First Affiliated Hospital of Xinjiang Medical University during the same period; (2) complete physical examination data; (3) can read, understand and sign the informed consent form.

Exclusion criteria for control group: (1) participants with any type of cancer or history of cancer;(2) patients with inflammatory diseases, such as pneumonia, urinary tract infections, or prostatitis 11.

The protocol was approved by the Ethics Committee of the First Hospital of Xinjiang Medical University (Approval No. 20220 308-166), and all study participants signed an informed consent form with a clear understanding of the purpose of the study protocol.

### 2.2 Data collection and measurements

Information regarding Participants’ general information (age), anthropometric measurements (including height, weight, and waist circumference), blood pressure, and blood samples were collected during standardized physical examinations. Participants were instructed to wear light clothing and remove their shoes for height and weight measurements. Waist circumference was measured by trained surveyors using a soft tape measure at the midpoint between the lower edge of the rib cage and the iliac crest. After a 10-minute rest period, blood pressure was measured using an upper-arm electronic sphygmomanometer. Following a 10–12 hour fasting period, blood samples were collected in the early morning *via* venipuncture from the antecubital vein. Fasting blood glucose(FBG), triglycerides(TG), HDL cholesterol(HDL-C), LDL cholesterol(LDL-C), serum creatinine(SCr), serum uric acid(SUA), monocyte count, neutrophil count, lymphocyte count, and platelet count were measured from the collected blood samples. All blood samples were analyzed enzymatically by trained laboratory technicians using an automated analyzer. Fasting blood glucose, triglycerides, and HDL cholesterol were measured using hexokinase, enzymatic, and clearance methods, respectively. Diabetes mellitus was defined as a fasting blood glucose level ≥126 mg/dL or current use of antidiabetic medication (32). Hypertension was defined as a systolic blood pressure ≥140 mmHg, a diastolic blood pressure ≥90 mmHg, or current use of antihypertensive medication (33, 34). Information on comorbidities, including coronary heart disease, was obtained from participants’ medical history records.

### 2.3 Calculation of the inflammation index

NLR, PLR, PIV, SIRI, and LMR were calculated using the neutrophil count (NEU), lymphocyte count (LYM), monocyte count (MON), and platelet count (PLT) obtained from the patient’s routine blood tests at the initial visit. According to the following formulas:


$$NLR=\frac{NEU}{LYM}$$

$$PLR=\frac{PLT}{LYM}$$

$$PIV=\frac{NEU\times PLT\times MON}{LYM}$$

$$SIRI=\frac{NEU\times MON}{LYM}$$

$$LMR=\frac{LYM}{MON}$$

### 2.4 Calculation of the CMI

The cardiometabolic index (CMI) was calculated as:


$$CMI=\left(\frac{TG}{HDL-C}\right)\times WHtR$$

Where WHtR (waist-to-height ratio) is Waist circumference (cm) /Height (cm).

### 2.5 Statistical analysis

Continuous variables were presented as mean ± standard deviation (SD) for normally distributed data or median with interquartile range (IQR; P25, P75) for skewed distributions. Group comparisons for continuous variables were performed using Student’s t-test (parametric) or Mann-Whitney U test (non-parametric) based on normality assessed via the Shapiro-Wilk test. Categorical variables were analyzed using Pearson’s χ² or Fisher’s exact test. To investigate the association between inflammatory indices, CMI index, and prostate cancer risk, we constructed multivariate logistic regression models with adjustments for potential confounders. The stability of these associations was verified through sensitivity analyses employing alternative variable categorization strategies and the exclusion of potential outliers. Nonlinear relationships were explored using generalized additive models (GAM) with cubic spline smoothing functions (3 degrees of freedom). Interaction effects between inflammatory indices were formally tested through GAM-based interaction models. Three-dimensional surface plots were generated to visualize interaction effects, where elevated Z-axis values (log-odds ratios) indicated stronger synergistic effects on prostate cancer risk. All statistical analyses were performed using R software (version 4.2.1; R Foundation for Statistical Computing) with two-tailed α level set at 0.05 for statistical significance. Smoothing functions in GAM were implemented using the mgcv package, and interaction plots were created using the plotly visualization toolkit.

## 3 Results

### 3.1 Baseline characteristics

A total of 445 study subjects were included by propensity score matching (PSM), of which 149 prostate cancer patients were in the case group and 296 in the control group. Height, monocyte count, lymphocyte count, HDL cholesterol, NLR, PLR, PIV, SIRI, LMR and CMI were compared between the two groups and the differences were statistically significant (*p* < 0.05). See **Table 1**.

**Table 1:** Analysis of baseline information.

Variables	Prostate cancer patients (n = 149)	Non-prostate cancer patients (n = 296)	Z/χ²	*p*
Age(years), M (Q_1_ , Q_3_ )	68.00 (62.00, 73.00)	66.00 (60.00, 72.00)	-1.91	0.057
Height (cm), M (Q_1_ , Q_3_ )	170.00 (168.00, 175.00)	172.00 (168.00, 176.00)	-2.04	0.041
Weight (kg), M (Q_1_ , Q_3_ )	75.00 (65.00, 80.00)	74.00 (68.00, 80.25)	-0.38	0.708
WC(cm), M (Q_1_ , Q_3_ )	90.00 (84.00, 95.00)	90.00 (85.00, 96.00)	-0.87	0.385
Monocyte (×10^9^/l), M (Q_1_ , Q_3_ )	0.49 (0.41, 0.64)	0.41 (0.34, 0.49)	-6.25	<0.001
Neutrophil (×10^9^/l), M (Q_1_ , Q_3_ )	3.56 (2.83, 4.73)	3.75 (3.03, 4.54)	-0.62	0.535
Lymphocyte (×10^9^/l), M (Q_1_ , Q_3_ )	1.55 (1.21, 2.10)	1.96 (1.61, 2.50)	-6.06	<0.001
Platelets (×10^9^/l), M (Q_1_ , Q_3_ )	220.00 (180.00, 257.00)	213.50 (176.75, 256.00)	-0.40	0.688
SCr(umol/L), M (Q_1_ , Q_3_ )	74.05 (62.00, 87.25)	79.70 (72.38, 89.80)	-3.97	<0.001
SUA(mmol/L), M (Q_1_ , Q_3_ )	299.70 (257.19, 375.12)	334.65 (284.80, 378.85)	-3.09	0.002
FBG(mmol/L), M (Q_1_ , Q_3_ )	5.37 (4.73, 6.58)	5.40 (5.06, 6.19)	-1.34	0.182
TG(mmol/L), M (Q_1_ , Q_3_ )	1.36 (1.05, 1.91)	1.27 (0.88, 1.83)	-1.30	0.194
HDL-C(mmol/L), M (Q_1_ , Q_3_ )	1.04 (0.83, 1.20)	1.15 (0.98, 1.32)	-4.72	<0.001
LDL-C(mmol/L), M (Q_1_ , Q_3_ )	2.69 (2.08, 3.18)	2.79 (2.21, 3.41)	-1.75	0.080
PIV, M (Q_1_ , Q_3_ )	251.21 (148.30, 431.51)	162.14 (104.47, 255.85)	-5.65	<0.001
NLR, M (Q_1_ , Q_3_ )	2.39 (1.59, 3.30)	1.87 (1.52, 2.32)	-4.36	<0.001
PLR, M (Q_1_ , Q_3_ )	137.62 (96.93, 200.00)	106.46 (86.38, 138.27)	-5.01	<0.001
LMR, M (Q_1_ , Q_3_ )	3.25 (2.38, 4.00)	4.97 (3.86, 6.11)	-10.39	<0.001
SIRI, M (Q_1_ , Q_3_ )	1.07 (0.75, 1.87)	0.77 (0.53, 1.10)	-6.49	<0.001
CMI, M (Q_1_ , Q_3_ )	0.71 (0.50, 1.07)	0.59 (0.35, 1.02)	-2.59	0.010
High blood pressure, n(%)			13.37	<.001
Yes	44 (29.53)	141 (47.64)		
No	105 (70.47)	155 (52.36)		
Diabetes, n(%)			0.30	0.585
Yes	58 (19.59)	26 (17.45)		
No	238 (80.41)	123 (82.55)		
Coronary heart disease, n(%)			1.28	0.258
Yes	19 (6.42)	14 (9.40)		
No	277 (93.58)	135 (90.60)		

Values represent Z-scores (Mann-Whitney U test) for continuous variables or χ² statistics for categorical variables, as appropriate based on data distribution.

### 3.2 Association between the inflammation index and CMI with PCa risk

The differences between CMI and inflammation indices (PIV, SIRI, LMR, PLR, NLR) were statistically significant in all three models (p < 0.001). In models 1 and 2, PIV, SIRI, PLR, and NLR were positively associated with PCa, and LMR was negatively associated with PCa. After fully adjusting for covariates (model 3), the pattern remained the same (PIV: OR=1.01, 95%CI:[1.01 ~ 1.01]; SIRI: OR=2.57, 95%CI:[1.86 ~ 3.54]; PLR: OR=1.01, 95%CI:[1.86 ~ 3.54]; PLR: OR=1.01, 95%CI. 95%CI:[1.01 ~ 1.01];NLR: OR=1.53,95%CI:[1.28 ~ 1.82];LMR: OR=0.48,95%CI:[0.40 ~ 0.57]). In model 1 without adjustment, the correlation between CMI and PCa was not statistically significant (p > 0.05); however, after adjusting for the covariates (model 2, model 3), CMI was significantly associated with PCa (p < 0.05). In model 3, for every 1-unit standard deviation increase in CMI, there was a 97% increase in the risk of PCa (OR=1.97, 95%CI:[1.38~ 2.81]. See **Table 2**.

**Table 2:** Inflammation index and CMI in relation to PCa risk.

Variables	Model 1		Model 2		Model 3	
	0R (95%CI)	*P*	0R (95%CI)	*p*	0R (95%CI)	*p*
PIV	1.01 (1.01~1.01)	<0.001	1.01 (1.01 ~ 1.01)	<0.001	1.01 (1.01 ~ 1.01)	<0.001
SIRI	2.45 (1.80 ~ 3.34)	<0.001	2.54 (1.85 ~ 3.49)	<0.001	2.57 (1.86 ~ 3.54)	<0.001
LMR	0.48 (0.40 ~ 0.56)	<0.001	0.47 (0.39 ~ 0.56)	<0.001	0.48 (0.40 ~ 0.57)	<0.001
PLR	1.01 (1.01 ~ 1.02)	<0.001	1.01 (1.01 ~ 1.01)	<0.001	1.01 (1.01 ~ 1.01)	<0.001
NLR	1.50 (1.26 ~ 1.78)	<0.001	1.53 (1.28 ~ 1.82)	<0.001	1.53 (1.28 ~ 1.82)	<0.001
CMI	1.34 (0.99 ~ 1.80)	0.060	1.58 (1.14 ~ 2.19)	0.006	1.97 (1.38 ~ 2.81)	0.001

Model 1 was unadjusted; Model 2 was corrected for age, hypertension, diabetes mellitus, and coronary artery disease based on Model 1; and Model 3 was further corrected for creatinine, uric acid, glucose, triglycerides, HDL, and LDL based on Model 2.

### 3.3 RCS curve assesses inflammatory index and CMI about PCa risk

**Figure 2:**
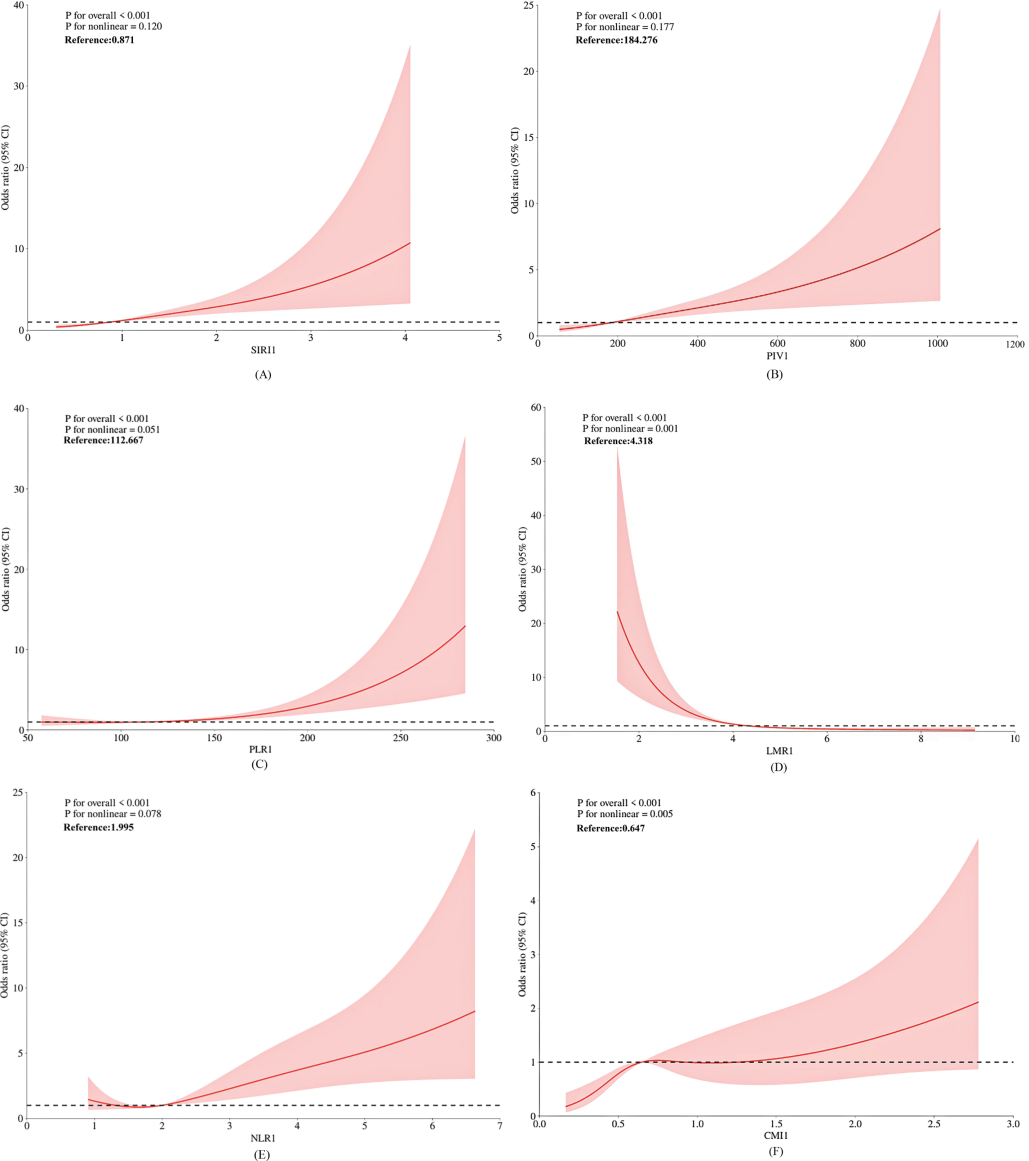
Dose-response relationships of inflammatory indices and CMI with prostate cancer risk.

The restricted cubic spline (RCS) model was constructed to evaluate the relationship between the inflammatory index, the cardiometabolic index (CMI), and the risk of PCa (**Figure 2**). As demonstrated in **Figures 2A-E**, sIRI () and PIV() exhibited a linear dose-response relationship with PCa risk (p for overall association < 0.001; p for nonlinearity > 0.05). PLR(), LMR(), NLR(), and CMI() demonstrated a nonlinear dose-response relationship with PCa risk (p for overall association < 0.001; p for nonlinearity < 0.05). As shown in **Figure 2F**, there is an initial sharp increase in prostate cancer risk in association with increasing CMI, and this increase attenuates once CMI is around 0.65.

### 3.4 Sensitivity analyses

Sensitivity analyses were performed to evaluate the robustness of the results. After excluding individuals with coronary heart disease, hypertension, and diabetes, the results consistently demonstrated significant correlations between PIV, SIRI, LMR, PLR, NLR, CMI, and prostate cancer. See **Tables 3**–**5**.

**Table 3:** Association between inflammatory indices and CMI with PCa risk after excluding coronary heart disease patients.

Variables	Model 1		Model 2		Model 3	
	0R (95%CI)	*p*	0R (95%CI)	*p*	0R (95%CI)	*p*
PIV	1.01 (1.01~1.01)	<0.001	1.01 (1.01 ~ 1.01)	<0.001	1.01 (1.01 ~ 1.01)	<0.001
SIRI	2.32 (1.70 ~ 3.16)	<0.001	2.41 (1.75 ~ 3.32)	<0.001	2.41 (1.74 ~ 3.33)	<0.001
LMR	0.48 (0.40 ~ 0.58)	<0.001	0.48 (0.40 ~ 0.57)	<0.001	0.49 (0.40 ~ 0.59)	<0.001
PLR	1.01 (1.01 ~ 1.02)	<0.001	1.01 (1.01 ~ 1.01)	<0.001	1.01 (1.01 ~ 1.01)	<0.001
NLR	1.47 (1.24 ~ 1.76)	<0.001	1.49 (1.24 ~ 1.78)	<0.001	1.49 (1.24 ~ 1.78)	<0.001
CMI	1.35 (0.99~1.84)	0.47	1.60 (1.14~2.24)	0.006	1.95 (1.35~2.80)	<0.001

Model 1 was unadjusted; Model 2 was corrected for age, hypertension, and diabetes mellitus based on Model 1; and Model 3 was further corrected for creatinine, uric acid, glucose, triglycerides, HDL, and LDL based on Model 2.

**Table 4:** Association between inflammatory indices and CMI with PCa risk after excluding hypertensive patients.

Variables	Model 1		Model 2		Model 3	
	0R (95%CI)	*P*	0R (95%CI)	*p*	0R (95%CI)	*p*
PIV	1.01 (1.01~1.01)	<0.001	1.01 (1.01 ~ 1.01)	<0.001	1.01 (1.01 ~ 1.01)	<0.001
SIRI	3.64 (2.22~ 5.95)	<0.001	3.62 (2.21 ~ 5.94)	<0.001	3.82 (2.29 ~ 6.35)	<0.001
LMR	0.44 (0.35 ~ 0.55)	<0.001	0.44 (0.35 ~ 0.56)	<0.001	0.44 (0.35 ~ 0.56)	<0.001
PLR	1.01 (1.01 ~ 1.02)	<0.001	1.01 (1.01 ~ 1.02)	<0.001	1.01 (1.01 ~ 1.02)	<0.001
NLR	1.74 (1.35 ~ 2.25)	<0.001	1.74 (1.34 ~ 2.26)	<0.001	1.79 (1.37 ~ 2.34)	<0.001
CMI	1.35 (0.86~2.11)	0.188	1.48 (0.93~2.36)	0.095	2.00 (1.19~3.35)	<0.009

Model 1 was unadjusted; Model 2 was corrected for age, coronary heart disease, and diabetes mellitus based on Model 1; and Model 3 was further corrected for creatinine, uric acid, glucose, triglycerides, HDL, and LDL based on Model 2.

**Table 5:** Association between inflammatory indices and CMI with PCa risk after excluding diabetic patients.

Variables	Model 1		Model 2		Model 3	
	0R (95%CI)	*p*	0R (95%CI)	*p*	0R (95%CI)	*p*
PIV	1.01 (1.01~1.01)	<0.001	1.01 (1.01 ~ 1.01)	<0.001	1.01 (1.01 ~ 1.01)	<0.001
SIRI	2.67 (1.87~ 3.81)	<0.001	2.74 (1.90 ~ 3.94)	<0.001	2.84 (1.94 ~ 4.15)	<0.001
LMR	0.45 (0.37 ~ 0.55)	<0.001	0.45 (0.37 ~ 0.55)	<0.001	0.45 (0.37 ~ 0.56)	<0.001
PLR	1.01 (1.01 ~ 1.02)	<0.001	1.01 (1.01 ~ 1.02)	<0.001	1.01 (1.01 ~ 1.02)	<0.001
NLR	1.63 (1.33 ~ 2.00)	<0.001	1.64 (1.34 ~ 2.02)	<0.001	1.65 (1.34 ~ 2.04)	<0.001
CMI	1.40 (0.95~2.06)	0.089	1.59 (1.05~2.39)	0.027	2.24 (1.42~3.52)	<0.001

Model 1 was unadjusted; Model 2 was corrected for age, coronary heart disease, and hypertension based on Model 1; and Model 3 was further corrected for creatinine, uric acid, glucose, triglycerides, HDL, and LDL based on Model 2.

### 3.5 Interaction of CMI with inflammation index

Concerning prostate cancer risk, the NLR exhibited a significant additive interaction with the CMI (RERI = 0.19, 95% CI: 0.01 to 0.37), indicating a potential synergistic effect. Nonetheless, further research is warranted to validate this additive interaction, given the non-significant results observed in the AP versus SI analysis (AP=0.14, 95%CI=-0.01~0.31; SI=2.45, 95%CI=0.03~203.91). See **Table 6**.

**Table 6:** Interaction of CMI with inflammation index.

Variables	Multiplicative scale (95%CI)	RERI (95%CI)	AP (95%CI)	SI (95%CI)
PIV	1.00 (0.99~1.00)	0.00 (-0.00~0.00)	0.00 (-0.00~0.00)	1.01 (0.89~1.14)
NLR	1.22 (0.88~1.68)	0.19 (0.01~0.37)	0.14 (-0.01~0.31)	2.45 (0.03~203.91)
PLR	1.00 (1.00~1.01)	0.00 (-0.00~0.00)	0.00 (-0.00~0.00)	–
LMR	0.71 (0.51~0.99)	-2.88 (-7.97~2.20)	-1.17 (-1.77~-0.56)	0.33 (0.22~0.51)
SIRI	1.24 (0.72~2.15)	0.48 (-0.11~1.09)	0.19 (-0.04~0.43)	1.46 (0.75~2.83)

**Figure 3:**
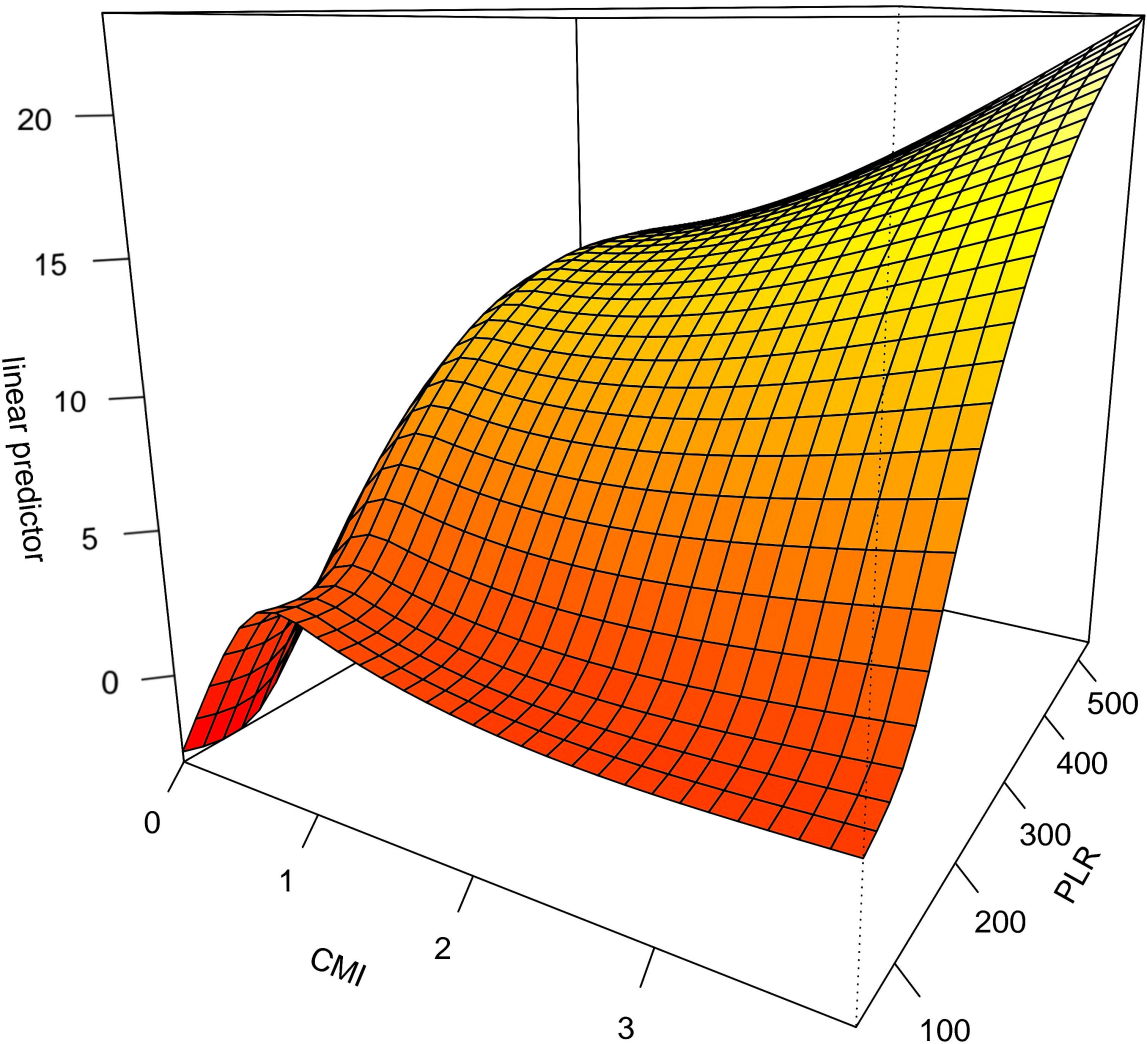
Interaction test on the basis of GAM between CMI and PLR on prostate cancer.

**Figure 4:**
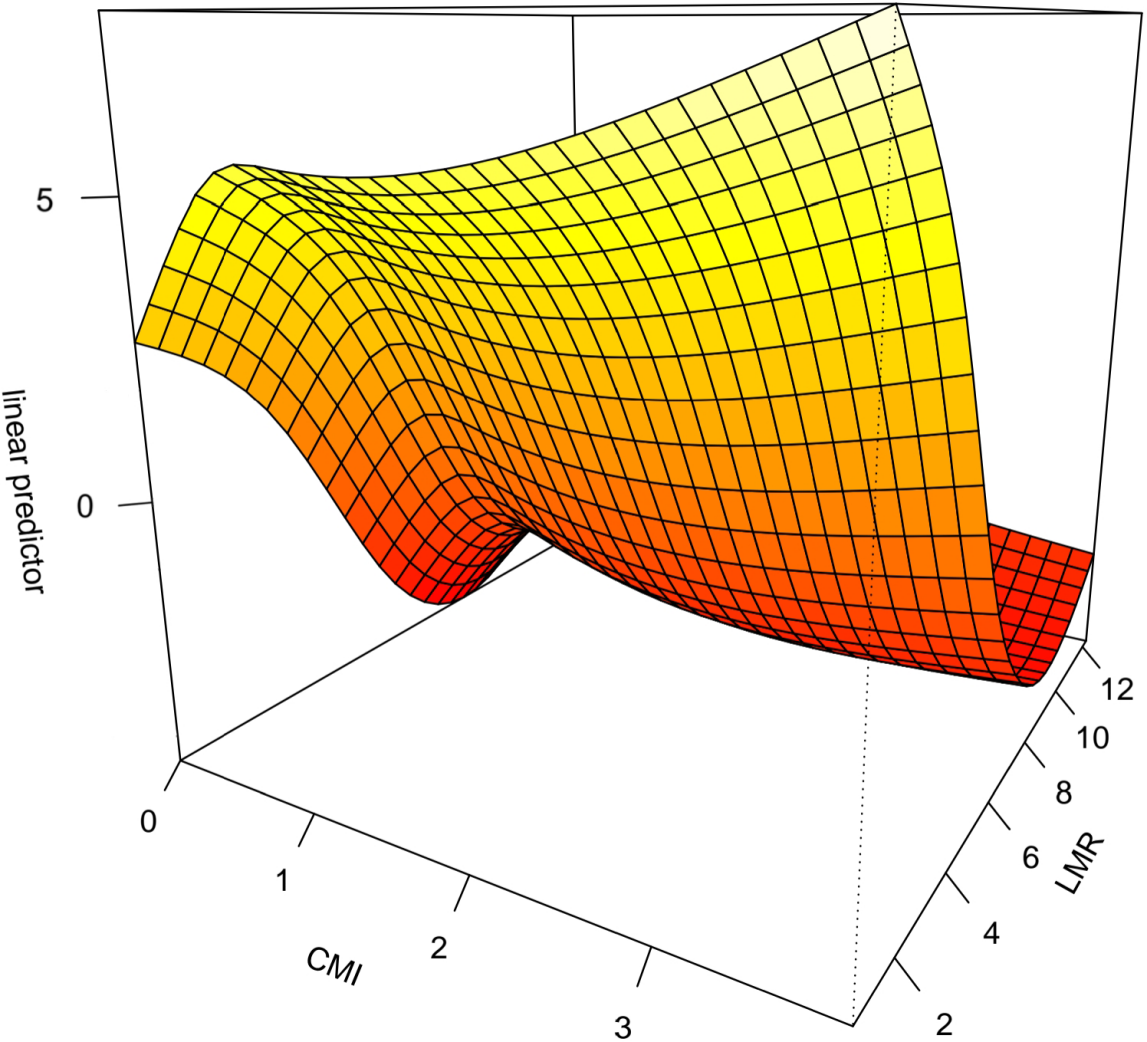
Interaction test on the basis of GAM between CMI and LMR on prostate cancer.

The interaction between CMI and different inflammatory factors in prostate cancer was assessed by constructing an interaction test and drawing a three-dimensional interaction model. There was a multiplicative interaction between PLR and CMI for prostate cancer (Multiplicative scale>1, p < 0.05).CMI > 3 and PLR > 400 were associated with the highest ratio (OR) for prostate cancer; the ratio (OR) for prostate cancer progressively decreased when CMI < 3 and PLR < 400. See **Figure 3**. There was a negative multiplicative interaction between LMR and CMI for prostate cancer (Multiplicative scale<1, p < 0.05). The highest ratio (OR) for prostate cancer was observed when CMI >3 and LMR <2. See **Figure 4**.

## 4 Discussion

Inflammation and obesity are two important risk factors for PCa (2)., Indeed, recent studies have demonstrated a significant association between inflammation and the cardiometabolic index (CMI) (29, 31). Furthermore, the cardiometabolic index (CMI) is a valuable marker for assessing obesity and lipid metabolism disorders (25–27) and has been strongly associated with various obesity-related diseases (35), as confirmed by multiple studies. In terms of obesity, adipose tissue, particularly visceral fat, serves as a significant source of chronic low-grade inflammation *in vivo*. This inflammation can promote PCa progression through mechanisms such as shaping the tumor inflammatory microenvironment and modulating lipid metabolism (28, 36). This suggests a possible complex link between inflammation, CMI, and PCa.

A chronic inflammatory state is strongly associated with the development of PCa (5, 37, 38). In this study, peripheral blood inflammatory indices were utilized to assess the systemic inflammatory status. The results demonstrated that NLR, PLR, SIRI, and PIV were significantly positively correlated with prostate cancer (PCa), whereas LMR exhibited a significant negative correlation. This finding is consistent with previous studies (20, 21, 39) and further confirms the correlation between inflammation and PCa. The mechanism by which inflammation leads to cancer development may be the inflammatory microenvironment established by inflammatory cells as well as various cytokines and chemokines (37). Cancer cells recruit and activate large numbers of leukocytes and other types of immune cells to infiltrate into the developing tumor site to induce inflammation (40). These immune cells facilitate tumor angiogenesis, invasion, metastasis, and proliferation (6). In the tumor microenvironment, the monocyte lineage promotes PCa cell progression by upregulating monocyte chemotactic protein-1 (MCP-1) expression and activating the nuclear factor-κB (NF-κB) signaling pathway (7). Neutrophils, on the other hand, accelerate tumor growth by releasing inflammatory cytokines, constructing an immunosuppressive microenvironment, and producing reactive oxygen species (ROS) to induce DNA damage (8). Furthermore, lymphopenia impairs anti-tumor immune surveillance, which leads to tumor immune escape and ultimately promotes tumor progression (9). Platelets and their metabolites promote tumor metastasis by influencing the coagulation cascade, activating oncogenic mutations, maintaining proliferative signals, and inducing angiogenesis (e.g., through the release of vascular endothelial growth factor) (10).

Numerous studies have demonstrated that obesity and lipid metabolism are strongly associated with tumorigenesis and metastasis (41). In this study, CMI was significantly associated with PCa risk. The risk of PCa increased rapidly with higher CMI levels. However, this trend plateaued when CMI reached approximately 0.65(see **Figure 2F**). This suggests that interventions targeting CMI may have greater clinical utility when CMI values are below 0.65.Elevated triglyceride levels and lowered high-density lipoprotein cholesterol (HDL-C) levels can lead to increased CMI, reflecting abnormal fatty acid metabolism (41). PCa cells may exploit these abnormal fatty acid metabolic pathways to generate energy and facilitate cell membrane synthesis, thereby promoting tumor growth and metastasis (36). Indeed, numerous studies have demonstrated an association between hypertriglyceridemia and the severity of PCa (42). Additionally, hypertriglyceridemia may reduce sex hormone-binding globulin (SHBG) levels, leading to increased free estrogen concentrations and further promoting PCa progression (43). Furthermore, reduced HDL levels and elevated LDL levels are associated with a poor prognosis in PCa patients (44), as the protective effects of HDL are diminished, while the tumor-promoting effects of LDL are enhanced (41).

The association between CMI and inflammation has been increasingly supported by numerous studies (29, 31). In our study, we found that the interaction between inflammatory indices and CMI affected the risk of PCa. The interaction between NLR, PLR, LMR, and CMI may synergistically promote tumor development by exacerbating inflammation, promoting angiogenesis, and suppressing anti-tumor immunity, respectively (45–49). Numerous studies have shown that inflammation is a key driver of obesity-related cardiometabolic diseases (50, 51). In obesity, the body typically exhibits chronic low-grade inflammation, which not only impairs vascular endothelial function and contributes to organ dysfunction but also triggers adipose tissue inflammation. This further exacerbates peripheral insulin resistance and amplifies the systemic inflammatory response via the release of cytokines and adipokines (51, 52). As a comprehensive indicator of cardiometabolic disease risk, CMI reflects visceral adiposity and lipid levels, particularly indicating central obesity (25). Central obesity can decrease plasma lipocalin, a hormone that inhibits angiogenesis and inflammation, by elevating pro-inflammatory adipokines. This reduction in lipocalin contributes to vascular and systemic inflammation (45, 47, 53). Additionally, central obesity plays a significant role in promoting tumor cell proliferation (54). Metabolic disorders reflected by CMI (e.g., abdominal obesity) release pro-inflammatory factors, while the inflammatory state itself activates immune cells to produce more inflammatory cytokines. Both act together to exacerbate the chronic low-grade inflammatory state (46). This enhanced chronic inflammation exacerbates the pro-inflammatory state of the tumor microenvironment and promotes tumor cell proliferation, survival, metastasis, and immune escape (46, 48). Furthermore, metabolic disorders and inflammation may promote tumor development by altering gene expression patterns through epigenetic modifications, which influence the expression of tumor suppressor genes and oncogenes (49).

In contrast to previous studies, the present study reveals the correlation between CMI and PCa, as well as elucidates the effect of the interaction between CMI and inflammatory indices on the risk of PCa. The value of CMI as a readily available indicator of visceral obesity for PCa was found, as well as the fact that CMI and inflammatory indices can work together as an adjunct tool to help in the prediction of PCa. These findings provide new perspectives for understanding the etiology of PCa; and provide important references for risk prediction and development of prevention strategies for PCa. Furthermore, it may pave the way for more refined screening and diagnostic strategies in the future.

## 5 Research limitations

This study has several limitations that need to be acknowledged. First, as a cross-sectional study, it is inherently limited in establishing causal relationships between the investigated variables and prostate cancer risk. Longitudinal studies are required to confirm temporal associations and to further explore the prognostic implications of these findings. Second, the additive interaction between NLR and prostate cancer requires validation in future studies, potentially due to the small sample size, which led to nonsignificant results for the attributable proportion (AP) and the confidence interval (CI). Future studies involving larger and more diverse populations are necessary to validate these findings. Third, although several potential confounders were adjusted for, residual confounding from unmeasured variables, such as lifestyle factors (e.g., diet, physical activity) and genetic predisposition, could not be entirely ruled out. Fourth, the absence of detailed data on prostate cancer subtypes, tumor stages, and treatment histories limited our ability to assess the impact of these factors on the observed associations. Finally, the single-center design of this study may have introduced selection bias and limited the generalizability of the findings. Multicenter studies are recommended to enhance the robustness and generalizability of the findings.

## 6 Conclusions

The results of this study demonstrated that PIV, SIRI, PLR, NLR, and CMI were positively associated with prostate cancer risk, whereas LMR exhibited a negative association. Furthermore, the findings revealed that the interaction between NLR, LMR, PLR, and CMI significantly influenced prostate cancer risk. These interactions suggest that inflammatory and metabolic factors may jointly contribute to prostate cancer development through distinct pathways, underscoring the importance of considering both inflammatory and metabolic status in prostate cancer risk assessment.

## Data Availability

The original contributions presented in the study are included in the article/supplementary material. Further inquiries can be directed to the corresponding authors.
